# Development of Automated Sleep Stage Classification System Using Multivariate Projection-Based Fixed Boundary Empirical Wavelet Transform and Entropy Features Extracted from Multichannel EEG Signals

**DOI:** 10.3390/e22101141

**Published:** 2020-10-09

**Authors:** Rajesh Kumar Tripathy, Samit Kumar Ghosh, Pranjali Gajbhiye, U. Rajendra Acharya

**Affiliations:** 1 Department of Electrical and Electronics Engineering, BITS-Pilani, Hyderabad Campus, Hyderabad 500078, India; rajeshiitg13@gmail.com (R.K.T.); samitnitrkl@gmail.com (S.K.G.); gajbhiyepranjali@gmail.com (P.G.); 2 School of Engineering, Ngee Ann Polytechnic, Singapore 599489, Singapore; 3 Department of Bioinformatics and Medical Engineering, Asia University, Taichung 41354, Taiwan; 4 School of Management and Enterprise, University of Southern Queensland, Springfield 4300, Australia

**Keywords:** entropy, sleep stages, multi-channel EEG, MPFBEWT, accuracy

## Abstract

The categorization of sleep stages helps to diagnose different sleep-related ailments. In this paper, an entropy-based information–theoretic approach is introduced for the automated categorization of sleep stages using multi-channel electroencephalogram (EEG) signals. This approach comprises of three stages. First, the decomposition of multi-channel EEG signals into sub-band signals or modes is performed using a novel multivariate projection-based fixed boundary empirical wavelet transform (MPFBEWT) filter bank. Second, entropy features such as bubble and dispersion entropies are computed from the modes of multi-channel EEG signals. Third, a hybrid learning classifier based on class-specific residuals using sparse representation and distances from nearest neighbors is used to categorize sleep stages automatically using entropy-based features computed from MPFBEWT domain modes of multi-channel EEG signals. The proposed approach is evaluated using the multi-channel EEG signals obtained from the cyclic alternating pattern (CAP) sleep database. Our results reveal that the proposed sleep staging approach has obtained accuracies of 91.77%, 88.14%, 80.13%, and 73.88% for the automated categorization of wake vs. sleep, wake vs. rapid eye movement (REM) vs. Non-REM, wake vs. light sleep vs. deep sleep vs. REM sleep, and wake vs. S1-sleep vs. S2-sleep vs. S3-sleep vs. REM sleep schemes, respectively. The developed method has obtained the highest overall accuracy compared to the state-of-art approaches and is ready to be tested with more subjects before clinical application.

## 1. Introduction

Sleep is one of the important activities of human beings and plays an important role in maintaining both mental and physical health [1,2]. Sufficient good quality sleep enhances the learning ability and performance of a person. Inadequate or a lack of proper sleep increases the occurrence of various sleep-related pathologies such as insomnia and bruxism, and other complications such as neurological diseases, cardiac diseases, hypertension, and diabetes [3]. Typically, sleep is categorized into wake, rapid eye movement (REM), and non-REM (NREM) sleep classes [4]. The sleep sub-types such as S1-sleep, S2-sleep, S3-sleep, and S4-sleep fall under the class of NREM sleep. The S1-sleep and S2-sleep sub-types are termed as light sleep (LS), whereas S3-sleep and S4-sleep sub-types are considered as deep sleep (DS) [5]. The heart activity, respiratory activity, eye movement, and muscle activity are slow during S1-sleep [6]. In S2-sleep, the eye movement is stopped, and there is also a drop in the body temperature. In the DS stage, the δ-wave activity of the brain increases, and heart rate and respiratory rate are dropped to the lowest level [7]. Moreover, during REM sleep, there is an increase in the physiological parameters such as blood pressure, heart rate, respiratory activity, and body temperature [8]. The rapid eye movements during this sleep stage affect the brain activity and these changes are faithfully reflected in the electroencephalography (EEG) signals of selected channels [7,9]. The polysomnography (PSG) test is normally performed in the clinical study for the diagnosis of sleep-related pathologies [10,11]. In the PSG test, various physiological signals such as EEG, electrocardiogram (ECG), respiratory signal, electromyogram (EMG), and oxygen saturation (${\mathrm{SPO}}_{2}$) are recorded from the subjects [1,3]. The human experts or sleep technologists manually assign the sleep classes to the segments of the physiological signal using Rechtschaffen and Kales (R& K) guidelines [12]. This process of sleep staging is cumbersome and, hence, automated approaches based on the analysis and classification of different physiological signals are needed. The discrimination of sleep stages from the physiological signal using one modality (e.g., EEG) can reduce the number of sensors used in the PSG test [13]. The multi-channel EEG signal has been used for the automated categorization of different sleep stages [9,14]. The development of a new approach for the automated categorization of various sleep stages using multi-channel EEG signals is an important research topic in neuroscience.

In the last two decades, different automated approaches have been employed for the automated categorization sleep stages using single-channel and multi-channel EEG signals [3,15,16,17]. A detailed review of the existing approaches is described in [18,19]. Song et al. [20] have used wavelet domain fractal analysis of single-channel EEG signal and quadratic discriminant analysis for the automated categorization of sleep stages. They have reported accuracies of 63.6%, 61.8%, 85.6%, and 21.7% for the classification of S1-sleep, S2-sleep, DS, and REM sleep categories, respectively. Similarly, Fraiwan et al. [21] have extracted Renyi entropy features in the time-frequency domains of single-channel EEG and used random forest classifier for the discrimination of different sleep stages. They have compared the performance of three time-frequency analysis methods such as Hilbert–Huang transform (HHT), Choi–Williams distribution (CWD), and discrete-time continuous wavelet transform (CWT) using EEG signals [21]. An overall accuracy score of 73.21% is reported for the categorization of S1-sleep, S2-sleep, S3-sleep, and REM sleep classes. Tsinalis et al. [22] have considered a convolutional neural network (CNN)-based deep learning approach for the automated categorization of sleep stages using single-channel EEG signals. They have reported an overall accuracy of 74% for the discrimination of S1-sleep, S2-sleep, S3-sleep, and REM sleep stage classes. Moreover, Huang et al. [14] have extracted spectral features from different bands of multi-channel EEG signals and used a multi-class support vector machine (MSVM) model to classify various sleep stages. Their method has reported an overall accuracy of 68.24%. Rodriguez-Sotelo et al. [9] have computed various non-linear features such as Shannon entropy, approximation entropy, sample entropy, detrended fluctuation analysis, multi-scale entropy, and fractal dimension features from two-channel EEG signals for the discrimination of sleep stages. They have used unsupervised learning method such as J-means clustering for the categorization of wake, S1-sleep, S2-sleep, S3-sleep, and REM sleep stages and obtained an accuracy of 57.4%. Moreover, Lagnef et al. [23] have extracted both time domain and spectral features from the multi-channel EEG signals and used a dendrogram-based SVM (DSVM) model for the categorization of wake, S1-sleep, S2-sleep, S3-sleep, and REM sleep types. They have achieved an overall accuracy of 74.8% using DSVM classifier. Andreotti et al. [24] have used CNN for the automated categorization of sleep stages using EEG signals from different databases. They have obtained a Kappa score value of 0.58 for five class sleep stage classification scheme using CNN. The CNN-based approach has demonstrated less classification performance compared to feature-based techniques using multi-channel EEG signals.

The existing approaches have used various uni-variate signal processing techniques for the classification of sleep stage classes with EEG signals. In recent years, various multivariate signal decomposition-based methods have been used for the analysis of different multi-channel physiological signals [1,25,26]. These methods considered all channel information of the physiological signals simultaneously for the decomposition. The multivariate empirical wavelet transform (MEWT) has been used for the categorization of seizure and seizure-free classes using multi-channel EEG signals [27]. In MEWT, the discrete Fourier transform (DFT) of individual channel EEG signal is computed and the average of DFTs of all EEG signals is used to generate the composite Fourier spectrum. The empirical wavelet filters are designed using the segments of composite Fourier spectrum [28]. The modes are evaluated using the designed wavelet filters and DFT of each channel EEG signal. The projection-based approach has been used in multivariate EMD (MEMD) and multivariate Fast and adaptive EMD (MFAEMD) to evaluate the composite signal from the multi-channel signal [29,30]. The advantage of the projection-based approach is that all channels are used to generate the composite signal. The parameters evaluated from the composite signal are used to extract the modes of each channel signal. The entropy measures have been widely used to quantify the information from EEG signals for various applications such as seizure detection, emotion recognition, and sleep stage classification [7,31,32]. The bubble entropy (BE)-based measure has been proposed for the analysis of heart rate variability (HRV) signals [33]. This measure used only one parameter such as an embedded dimension to quantify the regularity and complexity of a time series. Similarly, the dispersion entropy (DE)-based information measure has also been employed for the categorization of sleep stage classes using single-channel EEG signals [3,34]. Both BE and DE can be used in the multi-scale domain of multi-channel EEG signals for the categorization of sleep stages. The hybrid learning-based classifier has been considered for various applications, namely the detection of heart pathology such as congestive heart failure using electrocardiogram (ECG) signal features [35], and heart valve pathology detection using phonocardiogram (PCG) signal features [36]. This classification approach is distance-based and it does not use any weight updating rule based on the gradient descent algorithm like neural networks or deep learning methods. The number of training parameters is less in hybrid learning compared to the deep learning-based classifiers [35]. The hybrid learning classifier can be used for the automated categorization of different sleep stages using multi-scale entropy features extracted from the multi-channel EEG signals. The novelty of this work is the development of a multivariate multi-scale information–theoretic approach for the categorization of sleep stages using multi-channel EEG signals. The contributions of this paper are as follows:

(I) Novel multivariate projection-based fixed boundary empirical wavelet transform (MPFBEWT) is introduced for the multi-scale decomposition of multi-channel EEG signals.

(II) Two novel entropies (BE and DE) are used to extract the features in the multivariate multi-scale domain of multi-channel EEG signals.

(III) A hybrid learning-based classifier is employed for the categorization of sleep stages.

The remaining sections of this manuscript are organized as follows. In Section 2, the multi-channel EEG signals collected from the public database for the proposed classification task is described. The proposed approach for the categorization of sleep stages is explained in Section 3. In Section 4, experimental results and its discussion are presented. The conclusion of the paper is highlighted in Section 5.

## 2. Multi-Channel EEG Database

In this work, we have downloaded the multi-channel EEG signals from the cyclic alternation pattern (CAP) sleep database (capslbdb) to develop the proposed information–theoretic approach for sleep stage classification [37,38]. The database consists of PSG recordings of various physiological signals from 16 normal and 92 different sleep-related pathology subjects. In the CAP sleep database, the sampling frequency values are different for various multi-channel EEG signals. Few multi-channel EEG signals have the sampling frequency of 512 Hz, whereas other signals have sampling frequency values of 256 Hz, 200 Hz, and 128 Hz. In this work, we have selected 25 multi-channel EEG recordings with 512 Hz sampling frequency of each recording. Out of these 25 multi-channel EEG recordings, six recordings are normal (n), seven are insomnia (ins), and one each for sleep bruxism (brux), and sleep-disordered breathing (sdb) are considered. These 25 multi-channel EEG recordings are obtained from 15 male and 10 female subjects with age ranges of 64±18, and 48±14 years, respectively [37]. The gain parameters for each channel EEG signal are fixed to 32.76 [37]. The sleep stage annotation for each 30 s of physiological recordings is given in the CAP sleep database [37]. The symbols S0, S1, S2, S3, S4, and REM are the annotations used for wake (S0), S1-sleep, S2-sleep, S3-sleep, S4-sleep, and REM sleep stages. The recordings used in this work are denoted as, n1, n2, n3, n5, n10, n11, ins2, ins4, ins5, ins6, ins7, ins8, ins9, brux1, sdb3, and rbd1-rbd10, respectively. The n1, n2, n3, n5, n10, and n11 are denoted as the multi-channel EEG recordings for first, second, third, fifth, tenth, and eleventh subjects of normal classes. Similarly, rbd1-rbd10 are interpreted as the multi-channel EEG recordings for the first to the tenth subjects of the rbd class. In the CAP sleep database, a few recordings contain nine EEG channels (F2-F4, F4-C4, C4-P4, P4-O2, F1-F3, F3-C3, C3-P3, P3-O1, C4-A1). Moreover, a few recordings have five EEG channels (FP2-F4, F4-C4, C4-P4, P4-O2, C4-A1). The four common EEG channels such as P4-O2, C4-A1, F4-C4 and C4-P4 are selected in this work for all 25 recordings.

## 3. Method

**Figure 1:**
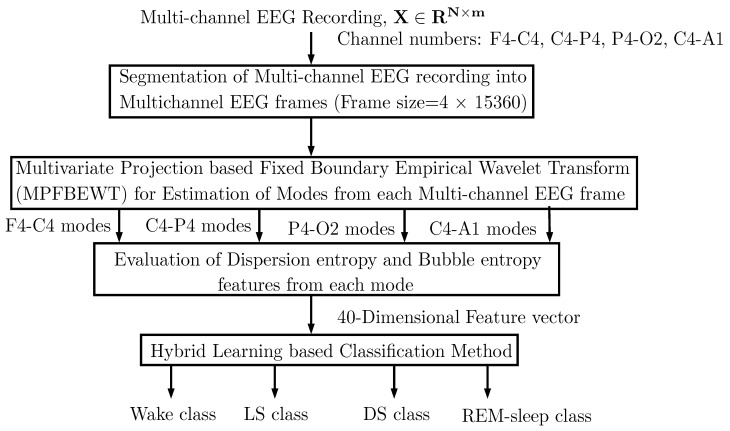
Flow chart showing the proposed automated approach for sleep stage classification.

The approach employed in this work for sleep stage classification is depicted in Figure 1. This approach consists of the evaluation of multi-channel EEG frames obtained from multi-channel EEG recordings using a segmentation technique. The multi-channel EEG frames are decomposed in to various sub-band signals using the MPFBEWT method. The BE and DE entropies are extracted from these sub-bands and clinically significant features are classified using hybrid learning classifier. Each stage involved in the flow chart is described in detail in the following sub-sections.

### 3.1. EEG Frame Evaluation

In this work, we have segmented each of the multi-channel EEG recordings into frames of 30 s duration. Before segmentation, the amplitude of each channel EEG signal is normalized by dividing the gain parameter of 32.76 [37]. The segmentation process is performed using a non-overlapping moving window of 30 s duration (15360 samples) [3]. In Table 1, we show the number of multi-channel EEG frames (or instances) used to evaluate the proposed approach for the automated discrimination of sleep stages.

### 3.2. Multivariate Fixed Boundary-Based EWT Filter Bank

The extension of EWT for the analysis of multi-channel signals is termed as the multivariate EWT [27]. The objective of EWT is the detection of boundary points in the Fourier spectrum of the analyzed signal [28]. Then, the contiguous segments extracted from the Fourier spectrum of the analyzed signal are used to design the empirical wavelet filter bank. In this work, we have proposed an MBFBEWT filter bank for the decomposition of multi-channel EEG signals. The sub-band signals of multi-channel EEG are evaluated in five steps. First, the multi-channel EEG signal $X\in R^{N\times m}$ is projected into a unit vector. The factor *m* is the total number of channels. In MFAEMD, the performance of the projection of a multi-channel signal X is based on the weighted sum of all channel signals [30]. For taking the projection, a point set for sampling on the (m−1)-dimensional unit sphere is considered [29]. The direction vector computed by a point on the (m−1)-dimensional unit sphere has the length *m*. The (m−1)-dimensional unit sphere contains the set of points ($v_{1}$, $v_{2}$, …, $v_{m}$) which satisfy the condition of $v_{1}^{2}+v_{2}^{2}+...+v_{m}^{2}=1$ in the Euclidean space. The vector representation of this point on (m−1)-dimension or channel unit sphere is given as $\hat{n}=v_{1}{\hat{n}}_{1}+v_{2}{\hat{n}}_{2}+\cdots \cdots \cdots +v_{m}{\hat{n}}_{m}$, where ${\hat{n}}_{1}$, ${\hat{n}}_{2}$………${\hat{n}}_{n}$ are the unit vectors of different channels [30]. In this study, we have considered the value of points such as ($v_{1}$, $v_{2}$, …, $v_{m}$) as the direction cosines for all channels and they are given by $\frac{1}{\sqrt{m}}$. This unit vector used in this work is given as follows [29,30]:

(1) $$\hat{n}=\frac{1}{\sqrt{m}}{\hat{n}}_{1}+\frac{1}{\sqrt{m}}{\hat{n}}_{2}+\cdots \cdots \cdots +\frac{1}{\sqrt{m}}{\hat{n}}_{m}$$

Similarly, the parameter *N* corresponds to the number of samples present in each channel of multi-channel EEG signal. The projected EEG signal is computed as follows:

(2) $${\mathrm{Pr}}_{\mathrm{EEG}}\left(n\right)=\frac{1}{\sqrt{m}}x_{{\mathrm{ch}}_{1}}\left(n\right){\hat{n}}_{1}+\frac{1}{\sqrt{m}}x_{{\mathrm{ch}}_{2}}\left(n\right){\hat{n}}_{2}+\cdots \cdots \cdots +\frac{1}{\sqrt{m}}x_{{\mathrm{ch}}_{m}}\left(n\right){\hat{n}}_{m}$$

where $x_{{\mathrm{ch}}_{1}}\left(n\right)$, $x_{{\mathrm{ch}}_{2}}\left(n\right)$, …, $x_{{\mathrm{ch}}_{m}}\left(n\right)$ are the EEG signals for different channels. In EWT, methods such as local maxima, scale-space, order statistics filter (OSF), etc., have been used for the detection of boundary points in the Fourier spectrum of the analysed signal [28,39]. For multivariate projection-based EWT, the filter bank can be designed based on the extraction of segments from the Fourier spectrum of the projected signal using any one of the boundary detection methods. However, in this study, the fixed boundary points are considered to design the filter bank. Hence, in the second step, we have considered a frequency grid as ($\left[-\frac{F_{s}}{2},\frac{F_{s}}{2}\right]$) instead of the DFT of the projected EEG signal for the creation of the filter bank [28]. Third, the fixed boundary points are evaluated to design the EWT filter bank. These boundary points are computed from the frequency points [40]. In this work, we have created an MBFBEWT filter bank using the frequency ranges of bands or rhythms of EEG signals. The δ, θ, α, β, and γ rhythms have frequency ranges such as 0–4 Hz, 4–8 Hz, 8–13 Hz, 13–30 Hz, and 30–75 Hz, respectively [41]. In this work, the frequency points $F=\left[4\;\;8\;\;13\;\;30\;\;75\right]$ are used to design the empirical wavelet filter bank [41]. The *t*th boundary point is obtained from the *t*th frequency point using the following relation [40]:

(3) $${\mathrm{FB}}_{t}=\frac{2\pi ⋆F_{t}}{F_{s}}$$

After obtaining the boundary points, the frequency grid ($\left[-\frac{F_{s}}{2},\frac{F_{s}}{2}\right]$) is segregated into segments for both positive and negative sides, and these segments are denoted as

(4) $$S_{t}=\left[{\mathrm{FB}}_{t-1}\;\;\;{\mathrm{FB}}_{t}\right]$$

where ${\mathrm{FB}}_{0}=0$, and ${\mathrm{FB}}_{N_{s}}=\frac{F_{s}}{2}$ [28]. The concatenation of all boundary points should cover the entire frequency range ($\left[0,\frac{F_{s}}{2}\right]$), and it is given by [28]

(5) $$\overset{N_{s}}{\underset{t=1}{⋃}}S_{t}=\left[0\;\;\;\frac{F_{s}}{2}\right]$$

where $N_{s}$ is the number of segments. In this work, a total of $N_{s}=6$ segments are extracted from the frequency domain representation of the projected EEG signal. In the fourth step, the empirical scaling and wavelet functions are used to create filters using the segments computed from the Fourier domain of the projected EEG signal. The empirical scaling function (SF) is given as follows [28]:

(6) $${\mathrm{SF}}_{t}=\left\{\begin{array}{c} 1;\;\;\mathrm{if}\;|k|\leq {\mathrm{FB}}_{t}-\eta_{t}\\ \cos\left[\frac{\pi }{2}g\left(\frac{1}{2\eta_{t}}\left(\left|k\right|-{\mathrm{FB}}_{t}+\eta_{t}\right)\right)\right];\;\;\\ \mathrm{if}\;\;{\mathrm{FB}}_{t}-\eta_{t}\leq \left|k\right|\leq {\mathrm{FB}}_{t}+\eta_{t}\\ 0;\;\;\mathrm{Otherwise} \end{array}\right.$$

Similarly, the empirical wavelet function (WF) is written as follows [28]:

(7) $${\mathrm{WF}}_{t}=\left\{\begin{array}{c} 1;\;\;{\mathrm{FB}}_{t}+\eta_{t}\leq \left|k\right|\leq {\mathrm{FB}}_{t}-\eta_{t}\\ \cos\left[\frac{\pi }{2}g\left(\frac{1}{2\eta_{t+1}}\left(|k\right|-{\mathrm{FB}}_{t+1}-\eta_{t+1})\right)\right];\;\;\\ \mathrm{if}\;{\mathrm{FB}}_{t+1}-\eta_{t+1}\leq \left|k\right|\leq {\mathrm{FB}}_{t+1}+\eta_{t+1}\\ \sin\left[\frac{\pi }{2}g\left(\frac{1}{2\eta_{t}}\left(|k\right|-{\mathrm{FB}}_{t}+\eta_{t})\right)\right];\\ \mathrm{if}\;{\mathrm{FB}}_{t}-\eta_{t}\leq \left|k\right|\leq {\mathrm{FB}}_{t}+\eta_{t}\\ 0;\;\;\mathrm{Otherwise} \end{array}\right.$$

The factor g(z) is given as $g\left(z\right)=35z^{4}-84z^{5}+70z^{6}-20z^{7}$ [28]. The transition phase width at *t*th boundary point is given as $2\eta_{t}$ [41]. The factor $\eta_{t}$ can be selected as $\eta_{t}=\alpha {\mathrm{FB}}_{t}$ where 0<α<1 [28]. The value of α is selected as $\alpha <\min_{t}\left(\frac{{\mathrm{FB}}_{t-1}-{\mathrm{FB}}_{t}}{{\mathrm{FB}}_{t+1}-{\mathrm{FB}}_{t}}\right)$ in order to get the sets $\left({\mathrm{SF}}_{1},{\left\{{\mathrm{WF}}_{t}\right\}}_{t=2}^{N_{s}}\right)$ as tight frames in the Euclidean space [28]. In the fifth step, the sub-band signals of the multi-channel EEG signal $x^{m}\left(n\right)$ are evaluated. The *m*th channel approximation sub-band signal is evaluated as follows:

(8) $$x_{1}^{m}\left(n\right)=R\left(\frac{1}{N}\sum_{k=0}^{N-1}{\mathrm{WS}}^{m}\left(k\right)e^{\frac{2\pi nk}{N}}\right)$$

where ${\mathrm{WS}}^{m}={\left[{\mathrm{WS}}^{m}\left(k\right)\right]}_{k=0}^{N-1}={\tilde{x}}^{m}{\bar{\mathrm{SF}}}_{1}$ is the frequency domain approximation sub-band signal and it is obtained using the multiplication of the spectrum of the *m*th channel EEG signal with the complex conjugate of the empirical scaling function [40]. The parameter $\bar{\mathrm{SF}}$ is termed as the complex conjugate of the scaling function. The ${\tilde{x}}^{m}={\left[{\tilde{x}}^{m}\left(k\right)\right]}_{k=0}^{N-1}$ is the DFT of the *m*th channel EEG signal $x^{m}={\left[x^{m}\left(n\right)\right]}_{n=0}^{N-1}$. Moreover, the *t*th detailed sub-band signal for the *m*th EEG channel is computed as follows [40]:

(9) $$x_{t=2,3\cdots \cdots N_{s}}^{m}\left(n\right)=R\left(\frac{1}{N}\sum_{k=0}^{N-1}{\mathrm{WW}}_{t}^{m}\left(k\right)e^{\frac{2\pi nk}{N}}\right)$$

where ${\mathrm{WW}}_{t}^{m}={\left[{\mathrm{WW}}_{t}^{m}\left(k\right)\right]}_{k=0}^{N-1}={\tilde{x}}^{m}{\bar{\mathrm{WF}}}_{t}$ is the frequency domain of the *t*th detailed sub-band signal obtained using the multiplication of the spectrum of the *m*th channel EEG signal with the complex conjugate of the empirical wavelet function for the *t*th segment [28]. The factor $\bar{\mathrm{WF}}$ is the complex conjugate of WF. Similarly, R(.) is denoted as the real part of the signal [28]. The algorithm for the evaluation of the sub-band signals of the *m*th channel is summarized in Algorithm 1.
**Algorithm 1:** Evaluation of modes obtained from multi-channel electroencephalogram (EEG) signal using multivariate projection-based fixed boundary empirical wavelet transform (MPFBEWT) filter bank.**Inputs:** Multi-channel EEG frame $X\in R^{N\times m}={\left[x^{m}\left(n\right)\right]}_{n=0}^{N-1}$, where *m* and *N* are the number of channels and samples, respectively.**Output:** A third order tensor, $Y\in R^{N\times m\times T}$, where *T* is the number of modes.**Step 1**: The multi-channel EEG signal is projected into a unit vector using Equation (2).**Step 2**: For a fixed boundary case, the frequency grid ($\left[-\frac{F_{s}}{2},\frac{F_{s}}{2}\right]$) is created. Similarly, the discrete Fourier transform (DFT) of the projected EEG signal can be evaluated for the automated boundary point evaluation case using local maxima or other methods.**Step 3**: Evaluate the fixed boundary points from the frequency points using Equation (3).**Step 4**: The EWT filter bank is created using the fixed boundary points of Step 3. The scaling and wavelet functions used to construct the EWT filter bank are mentioned in Equation (6), and Equation (7), respectively.**Step 5**: Evaluation of modes for *m*th channel of EEG signal using Equation (8), and Equation (9), respectively.

**Figure 2:**
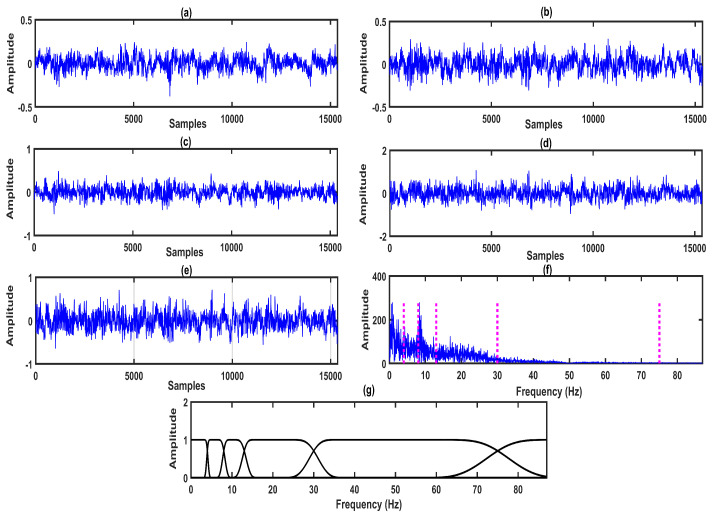
(**a**) EEG signal for F4-C4 channel. (**b**) EEG signal for C4-P4 channel. (**c**) EEG signal for P4-O2 channel. (**d**) EEG signal for C4-A1 channel. (**e**) Projected EEG signal. (**f**) Spectrum of projected EEG signal and Fixed frequency points. (**g**) MPFBEWT filter bank obtained from the spectrum of Projected EEG signal.

**Figure 3:**
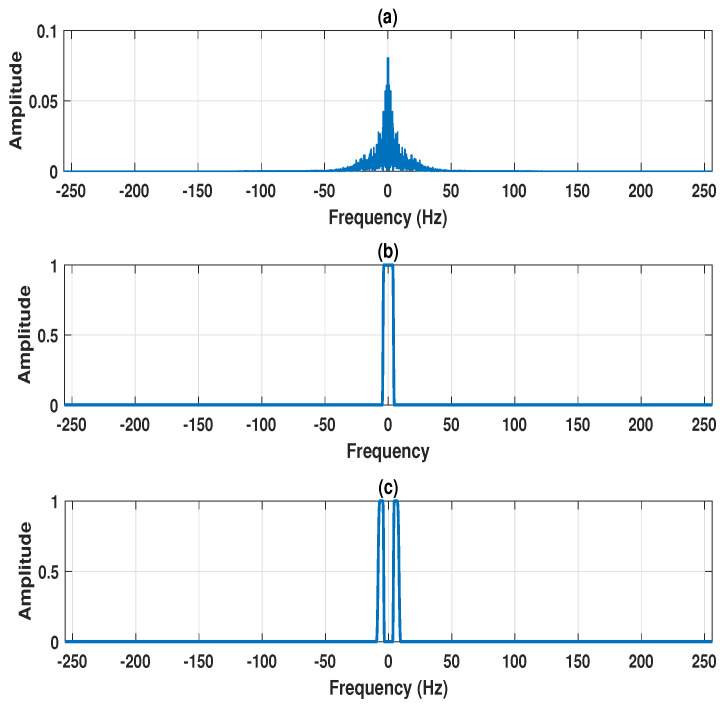
(**a**) Spectrum of projected EEG signal. (**b**) Frequency response of filter 1, created in the frequency range 0–4 Hz using empirical scaling function. (**c**) Frequency response of filter 2, created in the frequency range 4–8 Hz using empirical wavelet function.

**Figure 4:**
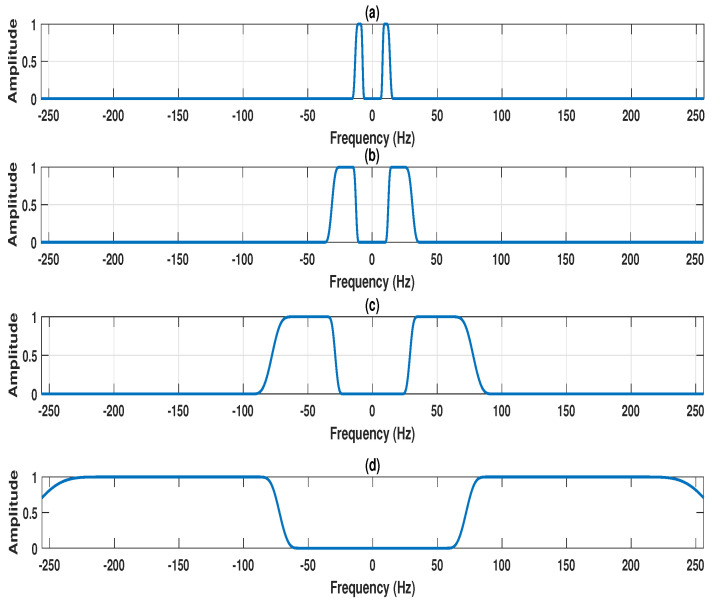
(**a**) Frequency response of filter 3, created in the frequency range 8–13 Hz using empirical wavelet function. (**b**) Frequency response of filter 4, created in the frequency range 13–30 Hz using empirical wavelet function. (**c**) Frequency response of filter 5, created in the frequency range 30–75 Hz using empirical wavelet function. (**d**) Frequency response of filter 6, created in the frequency range 75–256 Hz using empirical wavelet function.

The four-channel EEG signals (F4-C4 channel, C4-P4 channel, P4-O2 channel, and C4-A1 channel) are shown in Figure 2a–d. The projected EEG signal evaluated from the multi-channel EEG is depicted in Figure 2e. The detected frequency points for the design of the MPFBEWT filter bank in the spectrum of the projected EEG signal are shown in Figure 2e. The MPFBEWT filter bank was computed using empirical scaling and wavelet functions that are depicted in Figure 2f. The purpose of considering the spectrum of the projected EEG signal for deriving an empirical wavelet filter bank is given as follows. In multi-channel signal decomposition approaches like MEMD and MFAEMD, the composite signal is evaluated at the initial step by considering the information of all channel signals [29,30]. The mean envelope is computed from the composite signal using maxima–minima detection and the evaluation of upper and lower envelopes [29]. The mean envelope is used to obtain the modes of each channel signal at each iteration or until the stopping criteria is fulfilled. Motivated by these studies, we have considered the segments from the spectrum of the projected EEG signal for deriving the empirical wavelet filter bank. Furthermore, this filter bank is used for the evaluation of sub-band signals of each channel EEG signal.The two-sided Fourier spectrum of the projected EEG signal is depicted in Figure 3a. As the sampling frequency of the EEG signal is 512 Hz, the spectral energy is distributed between 0 and 256 Hz in both sides of the Fourier spectrum. The frequency domain scaling function obtained using Equation (7) for segment 1 is shown Figure 3b. It is observed that the scaling function is a low-pass filter with cut-off frequency value of 4Hz. Similarly, the wavelet functions obtained using Equation (8) for segment 2, segment 3, segment 4, segment 5, and segment 6 are shown in Figure 3c, Figure 4a–d, respectively.

**Figure 5:**
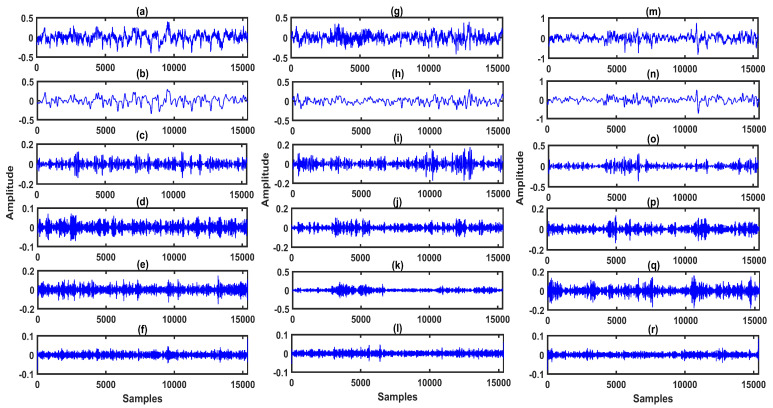
(**a**) EEG signal of F4-C4 channel for wake class. (**b**)–(**f**) Sub-band signals or rhythms (δ-wave, θ-wave, α-wave, β-wave, and γ-wave) of EEG signal extracted using MPFBEWT for wake class. (**g**) EEG signal of F4-C4 channel for S1 sleep class. (**h**)–(**l**) Sub-band signals or rhythms of EEG signal extracted using MPFBEWT for S1 sleep class. (**m**) EEG signal of F4-C4 channel for S2 sleep class. (**n**–**r**) Sub-band signals or rhythms of EEG signal extracted using MPFBEWT for S2 sleep class.

**Figure 6:**
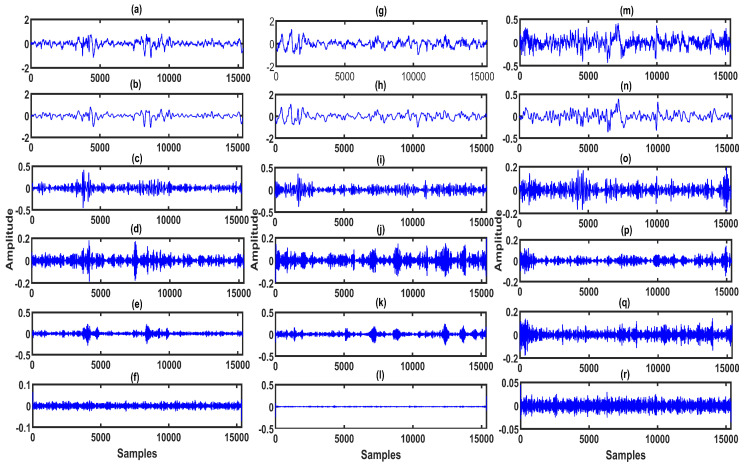
(**a**) EEG signal of F4-C4 channel for S3 sleep class. (**b**–**f**) Sub-band signals or rhythms (δ-wave, θ-wave, α-wave, β-wave, and γ-wave) of EEG signal extracted using MPFBEWT for S3 sleep class. (**g**) EEG signal of F4-C4 channel for S4 sleep class. (**h**–**l**) Sub-band signals or rhythms of EEG signal extracted using MPFBEWT for S4 sleep class. (**m**) EEG signal of F4-C4 channel for REM sleep class. (**n**–**r**) Sub-band signals or rhythms of EEG signal extracted using MPFBEWT for REM sleep class.

The F4-C4 channel EEG signals for the wake, S1-sleep, S2-sleep classes are depicted in Figure 5a,g,m, respectively. Similarly, for S3-sleep, S4-sleep, and REM sleep classes, F4-C4 channel EEG signals are shown in Figure 6a,g,m, respectively. The sub-band signals for the wake, S1-sleep, and S2-sleep classes are shown in Figure 5b–f and Figure 5h,i, and Figure 5n–r, respectively. Moreover, in Figure 6b–f and Figure 6h,i, and Figure 6n–r, we show the sub-band signals for S3-sleep, S4-sleep, and REM sleep stage classes. In the S1-sleep stage, the θ-wave activity increases in the EEG signal [42]. Similarly, in the early portion of S1-sleep stage, the α-waves are seen in the EEG signal [43]. Moreover, in S2-sleep, sleep spindles and K-complexes are present in the EEG signal. In S3-sleep and S4-sleep stages, the δ-wave activity increases in the EEG signal, and it is difficult to awaken a person during these sleep stages [42]. Furthermore, the REM sleep stage EEG signal characteristics are very similar to that of the wake stage EEG signal—this is the dreaming stage [42]. The muscle activity and the eye movements increase the amplitude of the EEG signal during the REM sleep stage [44]. As seen from the plots in Figure 5 and Figure 6, for different sleep stage classes, the characteristics of sub-band signals or rhythms of EEG are also different. These differences can be effectively captured by extracting the features from the sub-band signals. In this study, the BE and the DE measures are computed from each sub-band signal of multi-channel EEG.

### 3.3. Entropy Features Extraction

In this work, we have extended the theories of DE and BE for the analysis of multi-channel EEG signals in multi-scale domain. The DE of *t*th sub-band signal of the *m*th channel $x_{t}^{m}\left(n\right)$ is evaluated using six steps. First, the sub-band signal $x_{t}^{m}\left(n\right)$ is mapped into a new signal, $y_{t}^{m}\left(n\right)$ using a normal cumulative distribution function (NCDF). The value of $y_{t}^{m}\left(n\right)$ varies between 0 and 1. Second, a linear function is used to assign a decimal value or level with the relation as follows [34]:

(10) $$z_{t}^{m,a}\left(n\right)=\mathrm{round}\;\left(a•y_{t}^{m}\left(n\right)+0.5\right)$$

where $z_{t}^{m,a}\left(n\right)$ represents the *n*th sample of the mapped signal. The factor *a* stands for the *a*th level or decimal value. In DE, each sample of the mapped signal is assigned a decimal value. In the third step, the embedded vectors are extracted from the mapped signal $z_{t}^{m,a}\left(n\right)$ using the embedded dimension as *L*. The embedded vector is evaluated as follows:

(11) $${\tilde{z}}_{t,i}^{m,a}\left(n\right)=\left[z_{t,i}^{m,a},z_{t,i+d}^{m,a},\cdots \cdots z_{t,i+(L-1)d}^{m,a}\right]$$

where *i* represents the *i*th embedded vector and i=1,2,⋯⋯N−(L−1)d. The parameter *d* is the time delay. The fourth step is the assignment of the dispersive pattern (DP) for the *i*th embedded vector and it can be written as $\pi_{\left\{r_{0},r_{1}\cdots \cdots r_{L-1}\right\}}$, where each element of the *i*th embedded vector is given by $z_{t,i}^{m,a}=r_{0}$, $z_{t,i+d}^{m,a}=r_{1}$,…, $z_{t,i+(L-1)d}^{m,a}=r_{L-1}$ [34]. The number of possible DPs for the mapped signal, $z_{t}^{m,a}\left(n\right)$ is given as $a^{L}$ [34]. In the fifth step, the relative frequency or probability of each DP for the *t*th sub-band signal of the *m*th channel is given by

(12) $$P_{t}^{m}\left(\pi_{\left\{r_{0},r_{1}\cdots \cdots r_{L-1}\right\}}\right)=\frac{\mathrm{count}\;\mathrm{number}\;\mathrm{of}\;i\;\mathrm{for}\;\mathrm{which}\;{\tilde{z}}_{t,i}^{m,a}\;\mathrm{has}\;a\;\mathrm{DP}\;\pi_{\left\{r_{0},r_{1}\cdots \cdots r_{L-1}\right\}}}{N-(L-1)d}$$

where i=1,2,⋯⋯N−(L−1)d. In the last step, the DE of the *t*th sub-band signal of he *m*th channel EEG is evaluated and it is given as follows [34]:

(13) $${\mathrm{DE}}_{t}^{m}=-\sum_{\pi =1}^{a^{L}}P_{t}^{m}\left(\pi_{\left\{r_{0},r_{1}\cdots \cdots r_{L-1}\right\}}\right)\;\ln\left[P_{t}^{m}\left(\pi_{\left\{r_{0},r_{1}\cdots \cdots r_{L-1}\right\}}\right)\right]$$

Parameters such as the embedded vector length (*L*), time delay (*d*), and level (*a*) are used to compute the DE of each sub-band signal of the *m*th channel. In this work, we have considered *L* as 10, *d* as 1, and *a* as 2, respectively. In this work, a small value for *L* is selected in order to avoid the under sampling in the embedded vector.

BE is a recently proposed information quantification measure, and has advantages in that requires only few features from the time series [33]. The BE of the *t*th sub-band signal of the *m*th channel, $x_{t}^{m}\left(n\right)$, is evaluated in five steps. First, the embedding vectors from the *t*th sub-band signal of the *m*th channel are computed using Equation (12) [33]. Second, the ’L’ elements in the *i*th embedding vector are sorted in an ascending order and the number of swaps are counted. The number of swaps for the *i*th embedding vector are denoted as ${\mathrm{ns}}_{i}$. Third, a histogram of the swap vector (a vector containing the swaps of all embedding vectors) is evaluated, and it is normalized to obtain the probability. The probability for the *t*th sub-band signal of the *m*th channel is given as follows:

(14) $$P_{t}^{m}\left(b\right)=\frac{h_{t}^{m}\left(b\right)}{N-m+1}$$

Fourth, the Renyi entropy for *t*th sub-band signal of the *m*th channel is evaluated as follows [33]:

(15) $$E_{t}^{m,L}=-\log\left[\sum_{b=1}^{B}P_{t}^{m}\left(b\right)\right]$$

where *B* is the total number of bins. Similarly, the Renyi entropy is also calculated by considering the embedding dimension as L+1, and it is denoted as $E_{t}^{m,L+1}$. In the fifth step, the BE for the *t*th sub-band signal of the *m*th channel is evaluated as follows:

(16) $${\mathrm{BE}}_{t}^{m}=\frac{E_{t}^{m,L+1}-E_{t}^{m,L}}{\log\frac{L+1}{L-1}}$$

In this study, for each sub-band of all four channels of EEG signals, the DE and BE features are computed. Thus, 20 dimensional BE and DE feature vectors are created. Hence, the entropy feature vector, which consists of 40 features of multi-channel EEG signals, is formulated and used as an input to the hybrid learning classifier for the automated categorization of sleep stages. The following sub-section describes the working of the hybrid learning classifier.

### 3.4. Hybrid Learning based Classifier

In this work, the hybrid learning classifier is used to discriminate various sleep stages using entropy features obtained from the multi-channel EEG signal in a multi-scale domain. This classifier is designed based on the residual of the class-specific sparse representation method and nearest neighbor distances [35]. The description of hybrid learning for sleep stage classification is shown in Algorithm 2.
**Algorithm 2:** Hybrid learning classifier algorithm for classification of sleep stages.**Inputs:** Training feature matrix ($F_{tr}\in R^{I_{tr}\times q}$), training class label ($L_{tr}\in R^{I_{tr}}$), test feature matrix ($F_{te}\in R^{I_{te}\times q}$), number of nearest neighbors (nn), desired sparsity level (ρ).**Output:** Predicted class label, $L_{P}\in R^{I_{te}}$**Step 1**: The training feature matrix $F_{tr}$ is taken as a dictionary for the sparse representation of the test feature vector. The *r*th test instance $f_{r}$ can be written as $f_{r}=\alpha^{1}F_{tr}^{1}+\alpha^{2}F_{tr}^{2}+\cdots \cdots \alpha^{e}F_{tr}^{e}$ [35]. where $F_{tr}^{e}$ is the feature matrix for *e*th class. $\alpha^{1},\alpha^{2},\cdots \cdots \alpha^{e}$ are the class-specific sparse representation vectors.**Step 2**: In this step, the combined sparse representation vector $\alpha =\left[\alpha^{1},\alpha^{2}\cdots \cdots \alpha^{e}\right]$ is evaluated using the orthogonal matching pursuit (OMP) method as the optimization problem based on the fact that the minimization of $L_{0}$-norm $\alpha =\arg\min_{\alpha }{∥\alpha ∥}_{0}$ subjected to $f_{r}=\alpha F_{tr}$ is NP-hard [45].**Step 3**: The residual for the *e*th class is computed as ${\mathrm{Res}}^{e}={∥f_{r}-\alpha^{e}F_{tr}^{e}∥}_{2}$ [35].**Step 4**: In this step, the distances between $f_{r}$ and all training instances for the *e*th class are computed and these distances are given as ${\mathrm{dis}}^{e}\left(j\right)={∥f_{r}-f_{tr_{j}}^{e}∥}_{2}$. Then, the nearest distances for each class are selected. The median value of these distances for each class are evaluated, and they are given by $D^{e}=\mathrm{median}\left({\mathrm{dis}}^{e}\left(1:\mathrm{nn}\right)\right)$, where nn is the number of nearest neighbors for each class.**Step 5**: The residual and distance for each class are summed up and the total distance (TD) is computed. The total distance for the *e*th class is given by ${\mathrm{TD}}^{e}={\mathrm{Res}}^{e}+D^{e}$ [35].**Step 6**: The distance vector is evaluated as $\mathrm{TD}=[{\mathrm{TD}}^{1},{\mathrm{TD}}^{2}\cdots \cdots {\mathrm{TD}}^{e}]$. The predicted class label for each feature vector in the test feature matrix is computed as $l_{p}=\arg\;\min_{e}\mathrm{TD}$ [35]. For all test instances, the predicted class label vector is given as $L_{P}$.

The matrix evaluated using the entropy features from the multi-channel EEG frames is written as $F\in R^{I\times q}$, where *I* is denoted as the total number of multi-channel EEG frames. Similarly, the factor *q* is termed as the number of entropy features. We have used hold-out and 10-fold based cross-validation (CV) schemes to develop the hybrid learning classifier [3,40]. For hold-out CV, 60%, 10%, and 30% of instances are considered as the training, validation, and testing of the hybrid learning classifier. Similarly, for 10-fold CV, 90% of instances from the feature matrix are used for training and the remaining 10% are used for the testing of the hybrid learning classifier in each fold [3,40]. The training and test feature matrices for the classification are given as F_tr_, and F_te_, respectively. Similarly, the class labels for training and testing the multi-channel EEG instances are given as L_tr_, and L_te_, respectively. In this work, five classification strategies are considered to evaluate the classification results using the hybrid learning classifier. These strategies are wake vs. sleep, wake vs. REM, wake vs. LS class vs. Ds class vs. REM, wake vs. S1-sleep vs. S2-sleep vs. S3-sleep vs. REM, and wake vs. S1-sleep vs. S2-sleep vs. S3-sleep vs. S4-sleep vs. REM, respectively [3,17]. In order to evaluate the classification performance, the overall accuracy, accuracy for the individual class and the kappa score are used [36,46]. The Cohen kappa is evaluated using the following mathematical expression as [47],

(17) $$\kappa =\frac{P_{\mathrm{op}}-P_{\mathrm{tp}}}{1-P_{\mathrm{tp}}}$$

where $P_{\mathrm{op}}$ and $P_{\mathrm{tp}}$ are the observed and total probability values, respectively. The observed and total probability values are evaluated from the confusion matrix [48]. The confusion matrix table for a four-class sleep stage categorization is shown in Table 2.

The observed probability is evaluated as follows:

(18) $$P_{\mathrm{op}}=\frac{\sum_{i=1}^{4}C_{ii}}{\sum_{i=1}^{4}\sum_{j=1}^{4}C_{ij}}$$

Similarly, the total probability is computed using the individual probability values and it is written as follows [47]:

(19) $$P_{\mathrm{tp}}=P_{W}+P_{\mathrm{LS}}+P_{\mathrm{DS}}+P_{\mathrm{REM}}$$

where $P_{W}$, $P_{\mathrm{LS}}$, $P_{\mathrm{DS}}$, and $P_{\mathrm{REM}}$ are the probabilities for wake, LS, DS, and REM sleep classes. These probabilities are evaluated as follows:

$$\begin{array}{c} P_{W}=\frac{\sum_{j=1}^{4}C_{1j}}{\sum_{i=1}^{4}\sum_{j=1}^{4}C_{ij}}\times \frac{\sum_{i=1}^{4}C_{i1}}{\sum_{i=1}^{4}\sum_{j=1}^{4}C_{ij}}\\ P_{\mathrm{LS}}=\frac{\sum_{j=1}^{4}C_{2j}}{\sum_{i=1}^{4}\sum_{j=1}^{4}C_{ij}}\times \frac{\sum_{i=1}^{4}C_{i2}}{\sum_{i=1}^{4}\sum_{j=1}^{4}C_{ij}}\\ P_{\mathrm{DS}}=\frac{\sum_{j=1}^{4}C_{3j}}{\sum_{i=1}^{4}\sum_{j=1}^{4}C_{ij}}\times \frac{\sum_{i=1}^{4}C_{i3}}{\sum_{i=1}^{4}\sum_{j=1}^{4}C_{ij}}\\ P_{\mathrm{NREM}}=\frac{\sum_{j=1}^{4}C_{4j}}{\sum_{i=1}^{4}\sum_{j=1}^{4}C_{ij}}\times \frac{\sum_{i=1}^{4}C_{i4}}{\sum_{i=1}^{4}\sum_{j=1}^{4}C_{ij}} \end{array}$$

## 4. Results and Discussion

**Figure 7:**
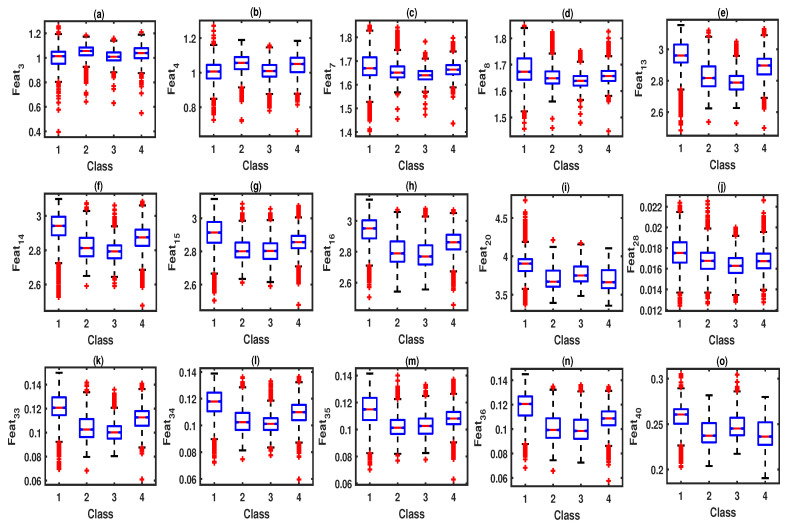
(**a**) Box-plot of ${\mathrm{Feat}}_{3}$ (dispersion entropy (DE) for 3rd sub-band of F4-C4 for wake (1), LS-class (2), DS-class (3), and REM sleep (4) classes. (**b**) Box-plot of ${\mathrm{Feat}}_{4}$ (DE for 1st sub-band of C4-A1) for all sleep stage classes. (**c**) Box-plot of ${\mathrm{Feat}}_{7}$ (DE for 3rd sub-band of C4-P4) for all sleep stage classes. (**d**) Box-plot of ${\mathrm{Feat}}_{8}$ (DE for 2nd sub-band of P4-O2) for all sleep stage classes. (**e**) Box-plot of ${\mathrm{Feat}}_{13}$ (DE for 3rd sub-band of C4-A1) for all sleep stage classes. (**f**) Box-plot of ${\mathrm{Feat}}_{14}$ (DE for 4th sub-band of F4-C4) for all sleep stage classes. (**g**) Box-plot of ${\mathrm{Feat}}_{15}$ (DE for 1st sub-band of C4-P4) for all sleep stage classes. (**h**) Box-plot of ${\mathrm{Feat}}_{16}$ (DE for 2nd sub-band of F4-C4) for all sleep stage classes. (**i**) Box-plot of ${\mathrm{Feat}}_{20}$ (DE for 4th sub-band of C4-P4) for all sleep stage classes. (**j**) Box-plot of ${\mathrm{Feat}}_{28}$ (bubble entropy (BE) for 3rd sub-band of P4-O2) for wake (1), LS-class (2), DS-class (3), and REM sleep (4) classes. (**k**) Box-plot of ${\mathrm{Feat}}_{33}$ (BE for 1st sub-band of C4-A1) for all sleep stage classes. (**l**) Box-plot of ${\mathrm{Feat}}_{34}$ (BE for 2nd sub-band of F4-C4) for all sleep stage classes. (**m**) Box-plot of ${\mathrm{Feat}}_{35}$ (BE for 3rd sub-band of C4-P4) for all sleep stage classes. (**n**) Box-plot of ${\mathrm{Feat}}_{36}$ (BE for 4th sub-band of P4-O2) for all sleep stage classes. (**o**) Box-plot of ${\mathrm{Feat}}_{40}$ (BE for 2nd sub-band of C4-A1) for all sleep stage classes.

This section shows the statistical analysis results of DE and BE features obtained from the sub-band signals of each EEG channel of wake, LS, DS, and REM sleep stages. The hybrid learning classifier results are shown for different classification schemes. A comparison with existing multi-channel based sleep stage classification approaches is also presented in this section. The box-plots of DE and BE features for different classes are shown in Figure 7. It can be observed from the statistical analysis results that eleven entropy features have shown higher mean values for the LS class. Similarly, five entropy features out of forty features have obtained higher mean values for the DS class. For the REM sleep class, three entropy features have shown higher mean values. Moreover, twenty-one entropy features have demonstrated higher mean values for the wake class. The θ-waves present in the EEG signal during LS have shown higher amplitude values compared to the α-waves [7,9]. Similarly, during the wake class, the EEG signal is irregular, and the neural activities are not synchronous. Furthermore, during DS stages, γ-wave patterns appear in the EEG signal [3]. Moreover, during REM sleep, the EEG signal morphology is different from EEG signals for wake and NREM sleep stage classes [3]. Due to these physiological changes in the EEG signals for different sleep stages, BE and DE features extracted in the multivariate multi-scale domain of multi-channel EEG signals have different mean values. The analysis of variance (ANOVA) test employed in this study confirms the statistical significance of entropy features for the automated categorization of sleep stages [49]. It can be seen from the ANOVA results that all multi-scale DE and BE features have p<0.001, and hence these entropy features are found to be clinically significant for the categorization of sleep stages using our proposed hybrid learning approach.

Table 3 shows the results obtained for proposed multivariate multi-scale approach for the automated categorization of the wake vs. sleep classification scheme with hold-out and 10-fold CV techniques using multi-channel frame selection techniques.

It is evident that the hybrid learning classifier has obtained accuracy and kappa scores of more than 91% and 0.80, respectively, for wake vs. sleep classification scheme (as shown in Table 3) using DE and BE features extracted from multi-channel EEG in the multi-scale domain. Similarly, the sensitivity and specificity values are also more than 90% for this classification scheme using the 10-fold CV strategy. Similarly, for hold-out CV, the hybrid learning classifier has obtained sensitivity, specificity and kappa score values of 86%, 91.16%, and 0.77, respectively. The classification results for wake vs. NREM vs. REM classification scheme using hold-out and CV methods are shown in Table 4.

It is seen that, the accuracy of the NREM class is higher than the accuracy of the REM and wake classes. The average kappa score of 0.74 is obtained using hybrid learning classifier with the 10-fold CV method. Our proposed method has yielded a higher performance with 10-fold CV compared to hold-out CV. Moreover, for the classification scheme such as wake vs. LS vs. DS vs. REM sleep, the results obtained using our method are depicted in Table 5.

It can be noted that the accuracy values of wake and deep sleep classes are more than 85% with the 10-fold CV method. The kappa score and overall accuracy values are higher for the 10-fold CV scheme compared to hold-out CV. The results of the classification task for wake vs. S1-sleep vs. S2-sleep vs. S3-sleep vs. REM using our proposed method are shown in Table 6.

It is evident that, for the wake, S2-sleep, S3-sleep, and REM sleep stage classes, the accuracy values are more than 72% with 10-fold CV. The S1 class has obtained the lowest accuracy using our proposed method. The average kappa score of 0.72 is obtained with 10-fold CV. Similarly, for the wake vs. S1-sleep vs. S2-sleep vs. S3-sleep vs. S4-sleep vs. REM classification scheme, the accuracy for each class, the kappa score, and the overall accuracy are shown in Table 7.

**Figure 8:**
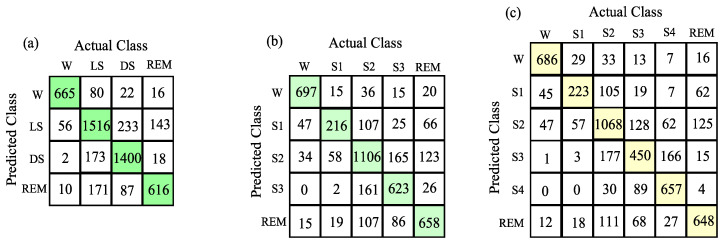
Confusion matrices obtained for various sleep classification schemes: (**a**) Wake vs. LS-class vs. DS-class vs. REM. (**b**) Wake vs. S1-sleep vs. S2-sleep vs. S3-sleep vs. REM. (**c**) Wake vs. S1-sleep vs. S2-sleep vs. S3-sleep vs. S4-sleep vs. REM.

It should be noted that the accuracy values of the proposed method are more than 80% for the wake and S4 classes using the 10-fold CV strategy. Similarly, for S2 and REM sleep classes, the accuracy values are more than 70%. The average kappa score value for the six-class sleep stage classification scheme using our method is 0.65. The confusion matrices obtained for the wake vs. LS-class vs. DS-class vs. NREM, wake vs. S1-sleep vs. S2-sleep vs. S3-sleep vs. REM, and wake vs. S1-sleep vs. S2-sleep vs. S3-sleep vs. S4-sleep vs. REM sleep stage classification schemes are shown in Figure 8a–c. It can be observed that the number of true positive percentages obtained for wake, LS, DS, and REM sleep classes are 84.92%, 77.82%, 87.88%, and 69.68%, respectively. Similarly, for wake, S1-sleep, S2-sleep, S3-sleep, and REM sleep stage classes, the true positive percentages obtained are 89.01%, 46.85%, 74.42%, 76.72%, and 74.35%, respectively. Moreover, the true positive percentages obtained for wake, S1-sleep, S2-sleep, S3-sleep, S4-sleep and REM classes are 87.5%, 48.37%, 71.82%, 55.41%, 84.23%, and 73.30%, respectively. These results clearly indicate that the DE and BE features successfully captured the information from multi-channel EEG recordings for the automated categorization of different sleep stage classes. Moreover, the classification results are also evaluated by varying the DE and BE parameters such as embedding vector length (L), time delay (d) and level (a). The overall accuracy and kappa score values obtained using the hybrid learning classifier for the wake vs. S1-sleep vs. S2-sleep vs. S3-sleep vs. S4-sleep vs. REM sleep classification scheme by varying DE and BE parameters are shown in Table 8.

In this work, the results are shown for both validation and test sets. It can be observed that the overall accuracy and kappa score values are 72.72%, and 0.637, respectively, for L = 10, a = 2, and d = 1 using feature vectors obtained from the multi-channel EEG frames of the test set. Similarly, overall accuracy and kappa score values of 72.18% and 0.631 are obtained using the feature vectors obtained from the validation set. Moreover, for other values of L, a, and d, the overall accuracy and kappa score are less for both test and validation sets. Hence, we have considered L = 10, a = 2, and d = 1 to compute DE and BE features from the sub-band signals of the multi-channel EEG signal.

We have selected hyper-parameters such as desired sparsity level (ρ) and the number of nearest neighbors (nn) of the hybrid learning classifier using the accuracy value of the validation set. The variations in the overall accuracy values with sparsity level and the number of nearest neighbors for the validation set and test set are shown in Table 9. It is observed that the hybrid learning classifier has an overall accuracy value of 37.96% for ρ = 2, and nn = 1, respectively. The overall accuracy value of the hybrid learning classifier increases by increasing the sparsity level from ρ = 2 to ρ = 20, and the nearest neighbors from nn = 1 to nn = 10, respectively. Moreover, the overall accuracy value decreases by increasing the sparsity level from ρ = 20 to ρ = 22, and the nearest neighbors from nn = 10 to nn = 11, respectively. Hence, the sparsity level of ρ = 20, and the number of nearest neighbors of nn = 10 are found as the optimal parameters for the hybrid learning classifier for the automated categorization of sleep stages.

Moreover, the proposed information–theoretic approach is compared with the existing multi-channel EEG based techniques for the categorization of wake vs. S1-sleep vs. S2-sleep vs. S3-sleep vs. REM sleep stages. Table 10 shows a summary of the results of a comparison with the state-of-art techniques. It can be observed from the reported works that the spectral features [14], and non-linear features [9], coupled with MSVM and J-means clustering techniques, have obtained lower overall accuracy values compared to the proposed MPFBEWT filter bank-based approach. The combination of both time domain and spectral features with DSVM classifiers led to higher overall accuracy values compared to our proposed information theoretic approach [23]. Moreover, the accuracy value reported using CNN-based transfer learning method is 67.70% [24], which is less than our proposed method. The proposed multivariate multi-scale approach has also demonstrated higher overall accuracy compared to the time-frequency domain Renyi entropy features combined with the random forest classifier [21]. The advantages of this study are summarized as follows:

(i) We obtained the highest classification performance compared to the spectral, and time–frequency-based entropy features of EEG signals.
(ii) The extracted discriminative multi-scale BE and DE entropy features have yielded high classification accuracy.
(iii) The proposed information–theoretic approach is simple and computationally less intensive.
(iv) The developed hybrid learning model is evaluated for five types of sleep stage classification strategies.
(v) We achieved a robust model using 10-fold CV and hold-out strategies.

The limitation of this work is that we used multi-channel EEG recordings obtained from only 25 subjects. In future, we intend to consider other entropy-based measures such as slope entropy [50], distribution entropy [51], state space domain correlation entropy [52,53], and other entropy measures [31] to improve the classification performance of sleep stages using more subjects.

## 5. Conclusions

A novel information–theoretic approach is proposed for the automated categorization of different sleep stage classes using multi-channel EEG signals. The approach is based on the decomposition of each channel EEG signal in to various sub-band signals using the MPFBEWT filter bank technique. The dispersion and bubble entropies are extracted from the sub-bands of the MPFBEWT filter bank. The classification of various sleep stages is performed using a hybrid learning classifier with these entropy features. Our proposed approach has obtained classification accuracy values of 91.77%, and 88.14% for wave vs. sleep, and wake vs. NREM vs. REM sleep categories. The classification results of the proposed approach can be further improved by using other entropy measures in the multi-scale domain of multi-channel EEG signals.

## Figures and Tables

**Table 1:** Number of multi-channel EEG frames considered for this work.

Classes	Wake	S1-Sleep	S2-Sleep	S3-Sleep	S4-Sleep	REM	Total
Number of Frames	2613	1537	4955	2707	2601	2947	17360

**Table 2:** Confusion matrix table for a four-class classification scheme.

True/Predicted	W	LS	DS	REM
**W**	$C_{11}$	$C_{12}$	$C_{13}$	$C_{14}$
**LS**	$C_{21}$	$C_{22}$	$C_{23}$	$C_{24}$
**DS**	$C_{31}$	$C_{32}$	$C_{33}$	$C_{34}$
**REM**	$C_{41}$	$C_{42}$	$C_{43}$	$C_{44}$

**Table 3:** Performance of proposed method for the automated categorization of wake vs. sleep classification scheme.

Cross-Validation	Accuracy (%)	Sensitivity (%)	Specificity (%)	Kappa Score
Hold-out CV	88.58±0.34	86.00±1.02	91.16±0.37	0.77±0.01
10-fold CV	91.77±0.91	90.93±1.79	92.61±1.59	0.83±0.01

**Table 4:** Performance of proposed approach for the automated categorization of wake vs. non-rapid eye movement (NREM) vs. rapid eye movement (REM) sleep stages.

Cross-Validation	Overall Accuracy (%)	Accuracy of Wake (%)	Accuracy of NREM (%)	Accuracy of REM (%)	Kappa Score
Hold-out CV	84.43±0.40	83.30±0.80	92.15±0.72	53.83±0.66	0.71±0.01
10-fold CV	88.14±0.73	84.61±1.95	94.26±0.70	66.77±2.62	0.74±0.01

**Table 5:** Performance of proposed approach for the automated categorization of wake vs. LS class vs. DS class vs. REM sleep stages.

Cross-Validation	Overall Accuracy (%)	Accuracy of Wake (%)	Accuracy of LS (%)	Accuracy of DS (%)	Accuracy of REM (%)	Kappa Score
Hold-out CV	76.01±0.43	84.37±0.57	72.87±0.67	80.90±1.05	55.58±1.70	0.66±0.00
10-fold CV	80.13±0.71	85.07±1.58	76.87±1.23	87.96±1.69	68.81±2.74	0.72±0.00

**Table 6:** Performance of proposed approach for the automated classification of wake vs. S1-sleep vs. S2-sleep vs. S3-sleep vs. REM.

Cross-Validation	Overall Accuracy (%)	Accuracy of Wake (%)	Accuracy of S1-Sleep (%)	Accuracy of S2-Sleep (%)	Accuracy of S3-Sleep (%)	Accuracy of REM (%)	Kappa Score
Hold-out	73.59±0.56	85.53±1.15	42.99±1.71	77.27±1.25	63.55±0.97	60.01±1.92	0.62±0.00
10-fold	73.88±1.48	87.02±1.46	48.86±3.80	74.81±1.74	74.28±3.23	73.36±1.59	0.65±0.01

**Table 7:** Classification performance of proposed approach for the automated categorization of wake vs. S1-sleep vs. S2-sleep vs. S3-sleep vs. S4-sleep vs. REM.

Cross-Validation	Overall Accuracy (%)	Accuracy of Wake (%)	Accuracy of S1-Sleep (%)	Accuracy of S2-Sleep (%)	Accuracy of S3-Sleep (%)	Accuracy of S4-Sleep (%)	Accuracy of REM (%)	Kappa Score
Hold-out	71.08±0.37	85.05±0.79	45.79±0.29	75.24±0.90	58.30±0.96	59.71±2.18	72.51±0.52	0.61±0.00
10-fold	71.68±0.84	86.64±2.00	48.33±3.29	72.59±1.69	57.22±2.25	82.42±2.19	72.88±2.59	0.65±0.01

**Table 8:** Variation of overall accuracy (%) and kappa scores obtained for Wake vs. S1-sleep vs. S2-sleep vs. S3-sleep vs. S4-sleep vs. REM classification scheme by varying embedding vector length (L), time delay(d) and level (a) parameters of DE and BE features.

Parameters			Validation Set		Test Set	
L	a	d	Overall Accuracy (%)	Kappa score	Overall Accuracy (%)	Kappa score
10	2	1	72.18	0.631	72.72	0.637
10	3	2	70.77	0.619	71.34	0.622
8	2	1	71.62	0.630	71.77	0.631
8	3	2	69.90	0.608	69.70	0.605
5	2	1	59.48	0.466	62.77	0.510
5	3	2	51.30	0.372	48.31	0.336

**Table 9:** Variations in overall accuracy values of hybrid learning classifier with desired sparsity level (ρ) and the number of nearest neighbors (nn) for validation and test sets for wake vs. S1-sleep vs. S2-sleep vs. S3-sleep vs. S4-sleep vs. REM classification scheme.

Sparsity Level	Nearest Neighbors	Overall Accuracy (%)	
ρ	**nn**	**Validation Set**	**Test Set**
2	1	37.96	39.78
4	2	56.11	56.87
6	3	60.67	62.08
8	4	65.38	65.97
10	5	65.62	68.04
12	6	69.38	70.11
14	7	69.70	71.01
16	8	70.12	70.97
18	9	70.18	72.48
20	10	72.18	72.72
22	11	71.54	71.66

**Table 10:** Comparison of our proposed method with existing techniques for the categorization of wake vs. S1-sleep vs. S2-sleep vs. S3-sleep vs. REM sleep stages using multi-channel and single-channel EEG signals.

Feature Extraction Methods	Classifier Used	Overall Accuracy (%)
Spectral Features evaluated from different rhythms of multi-channel EEG signals [14]	MSVM	68.24
Different non-linear features extracted from multi-channel EEG signals [9]	Unsupervised learning (J-means clustering)	57.40
Time domain and spectral features extracted from multi-channel EEG [23]	DSVM	74.80
Learnable features evaluated from multi-channel EEG signal in convolution layer stages [24]	Transfer learning using CNN	67.70
Renyi entropy features computed from the time-frequency representation of single-channel EEG signals [21]	Random forest	73.21
Multi-scale DE and BE features extracted from Multi-channel EEG signal (proposed work)	hybrid learning	73.88
